# In vitro antihypertensive and antioxidative properties of trypsin‐derived *Moringa oleifera* seed globulin hydrolyzate and its membrane fractions

**DOI:** 10.1002/fsn3.826

**Published:** 2018-11-25

**Authors:** Taiwo Ayodele Aderinola, Tayo Nathaniel Fagbemi, Victor Ndigwe Enujiugha, Adeola Monisola Alashi, Rotimi Emmanuel Aluko

**Affiliations:** ^1^ Department of Food Science and Technology The Federal University of Technology, Akure Akure Nigeria; ^2^ Department of Food and Human Nutritional Sciences University of Manitoba Winnipeg Manitoba Canada

**Keywords:** ACE, antihypertensive, globulin, hydrolyzate, membrane fractions, *Moringa oleifera* seed, renin

## Abstract

*Moringa oleifera* seed globulin was hydrolyzed with trypsin and fractionated to produce <1, 1–3, and 3–5 kDa peptide sizes. These were evaluated for antioxidant properties: DPPH, hydroxyl radical scavenging assays, FRAP, and metal chelation tests; and in vitro antihypertensive properties: ACE and renin inhibition. Membrane fractionation led to improved antioxidative properties of 29.13% (<1 kDa), 180% (<1 kDa), and 30.58% (1–3 kDa) for DPPH, FRAP, and metal iron chelation, respectively. There was however 48.77% reduction (1–3 kDa) in hydroxyl radical scavenging activity. There was also improvement in ACE inhibitory potentials of the peptides with the 1–3 kDa peptide showing significantly highest ACE inhibition (72.48%)but very low (17.64%, 1–3 kDa) inhibition against the renin. It was concluded that hydrolysis of M oleifera seed globulin with trypsin produced peptides and peptide fractions with potential antioxidant and antihypertensive properties.

## 1 INTRODUCTION

*Moringa oleifera* is a popularly grown plant in many tropical and subtropical countries including India, Thailand, Philippines, Pakistan, Nigeria, and Senegal (Anwar, Zafar, & Rashid, 2006). The multipurpose nature of the plant, including usage in traditional folklore medicine, animal feeds, spices, and other different preparations for human consumption, has contributed immensely to its wide acceptance and popularity (Ajibola, Fashakin, Fagbemi, & Aluko, 2011). Previous studies had reported the high nutritional values and varied health benefits of *M. oleifera*. Detailed potency of its ability against bacterial, fungal, epilepsy, diabetes, hypertension, ulcer, and inflammation is well documented as well as its hepatoprotective, antipyretic, and cholesterol‐lowering abilities (Ajibola, Fashakin, Fagbemi, & Aluko, 2013; Ajibola et al., 2011; Akinyede, Girgih, Osundahunsi, Fagbemi, & Aluko, 2016).

In the past two or three decades, many research had focussed on exploring the varied nutritional and health benefits of *M. oleifera* plant from leaf to the roots (Anhwange, Bjibola, & Oniye, 2004; Dollah, Abdulkarim, Ahmad, Khoramnia, & Ghazali, 2015; Mbah, Eme, & Ogbusu, 2012; Tesfay, Bertling, Odindo, Workneh, & Mathaba, 2011). While the traditional use of many plant species to treat diseases or ailment is widely practiced globally (Cumby, Zhong, Naczk, & Shahidi, 2008), there is a lack of proper documentation of such knowledge, or in some cases, they are only verbally transferred from one generation to the other. Therefore, consumption of foods, such as *M. oleifera* with established disease‐prevention abilities besides their basic nutritional functions, is currently the focus of many research studies (Ajibola, Malomo, Fagbemi, & Aluko, 2016; Akinyede et al., 2016; Oosthuizen et al., 2018).

The ability of food proteins or peptide fractions to impart further health benefits beyond their basic roles of supplying the body with the needed nutrition had been reported (Bekhit, Carne, & Birch, 2014; Doyen et al., 2014; He, Girgih, Malomo, Ju, & Aluko, 2013). *Moringa oleifera* is still an underutilized plant with much potential yet to be explored. While many positive health benefits have been attributed to *M. oleifera*, most of such reports were based on phenolic extracts of the different parts of the plant (Ajibola et al., 2011; G. H. Li, Le, Liu, & Shi, 2005), and there is very little information on the health benefits with respect to antioxidant and antihypertensive properties of the plant's (seed) proteins and fractions. Therefore, the potential of trypsin and subsequent membrane fractionation to release peptide fractions with antioxidant and antihypertensive properties was evaluated in this study.

## 2 MATERIALS AND METHODS

### 2.1 Material source

*Moringa* seeds were purchased from Oba market, Akure, Ondo State, Nigeria. Trypsin, 1,1‐diphenyl‐2‐picrylhydrazyl (DPPH), glutathione (GSH), and other antioxidant reagents were purchased from Sigma‐Aldrich (St. Louis, MO, USA). Other analytical grade reagents were purchased from Fisher Scientific (Oakville, ON, Canada).

### 2.2 Sample preparation

*Moringa* seed flour was obtained from the seed by grinding the seed with coffee grinder (Cuisinart, DCG 12BCC, China). It was defatted with acetone (1:10 w/v) under a continuous stirring for 1 hr allowed to sediment, filtered, and re‐dispersed in the same volume of acetone and the process was repeated for another 1 hr. After decanting off the excess acetone, it was air‐dried under fume cupboard and ground to obtain the *Moringa oleifera* seed meal (MSM).

### 2.3 Production of protein globulin

*Moringa* seed protein globulin was obtained through the process of dialysis as described by Aluko (2004) with some modifications. Briefly, MSM was dispersed in 0.5 M NaCl for 1 hr with continuous stirring followed by centrifugation (8,000 g, 60 min at 4°C). The supernatant was clarified with Whatman No 1 filter paper and the residue discarded. The filtrate was dialyzed for 5 days at 4°C using the 6–8 kDa MWCO dialysis tubing, and the dialysis water was changed at least three times daily. Thereafter, the content of the dialysis tube was centrifuged (8,000 g, 60 min at 4°C). The precipitate was washed with distilled water, centrifuged under similar conditions and collected as the globulin protein fraction (GPF). This was freeze‐dried, and the protein contents were determined using Kjeldahl method.

### 2.4 Hydrolysis of globulin protein fraction (GPF)

*Moringa* protein hydrolyzate (MPH) was obtained from the GPF. Hydrolysis of the GPF was conducted using trypsin (37°C, pH 8) as previously reported (He, Alashi et al., 2013). GPF (4%, w/v, protein basis) was dispersed in deionized water in a beaker, stirred continuously, heated to the appropriate temperature, and adjusted to the appropriate pH value as stated above prior to addition of the enzymes. Enzyme was added to the GPF slurry at 4% (mg/g) an enzyme to substrate based on the protein content of the substrate. Digestion was performed for 3 hr (pH maintained constant by addition of 1 M NaOH or 1 M HCl) after which the enzyme was inactivated by adjusting to pH 5.0 with 1 M HCl or 1 M NaOH heated at 95°C in a water bath for 15 min and cooled rapidly in an ice bath. The undigested proteins were precipitated by centrifugation at 8000× g for 60 min. A portion of the supernatant containing target peptides was freeze‐dried as MPH.

### 2.5 Methods

#### 2.5.1 Determination of DPPH radical scavenging activity

The scavenging activity of samples against the DPPH radical was determined using a previously described method (He, Alashi et al., 2013) with slight modifications for a 96‐well clear flat‐bottom plate. Samples were dissolved in 0.1 M sodium phosphate buffer, pH 7.0 containing 1% (w/v) Triton X‐100. DPPH was dissolved in methanol to a final concentration of 100 μM. Peptide samples (100 μl) at 1 mg/ml final assay concentration were mixed with 100 μl of the DPPH solution in the 96‐well plate to a final assay concentration of 1 mg/ml and incubated at room temperature in the dark for 30 min. The absorbance values of the control (*A*_*c*_) and samples (*A*_*s*_) were measured at 517 nm. The control consisted of buffer in place of the peptide sample while GSH was used as the positive control. The percentage DPPH radical scavenging activity of the samples was determined using the following equation:

$$\text{DPPH Radical Scavenging Activity}\left(\%\right)=\frac{A_{c}-A_{s}}{A_{c}}$$

where *A*_*c*_ and *A*_*s*_ are the absorbance of control and sample, respectively.

#### 2.5.2 Determination of ferric reducing antioxidant power (FRAP)

The ferric reducing antioxidant power of samples was measured according to a previously described method (Mundi & Aluko, 2014) with some modifications for a microplate reader. Briefly, the FRAP reagent was freshly prepared by mixing 300 mM acetate buffer (sodium acetate buffer, pH 3.6), 10 mM 4,6‐tripryridyls‐triazine (TPTZ) in 40 mM HCl, and 20 mM ferric chloride in a ratio 5:1:1 (v/v) before evaluation. Two hundred microliters (200 ml) of FRAP reagent (preheated to 37°C) was added to 40 μl of sample or GSH in a 96‐well microplate. Absorbance at 593 nm was measured relative to a reagent blank. Ferrous sulfate (conc: 0.0625–1 mM) was used to prepare a standard curve, and the results of the samples were expressed in mmol FeSO4.

#### 2.5.3 Determination of chelation of metal ions

The metal chelating activity was measured using a modification of a previously reported method (Girgih, Udenigwe, & Aluko, 2011). Five hundred microliter (500 μl) peptide sample solution or GSH (final assay concentration of 1 mg/ml) was combined with 25 μl of 2 mM FeCl_2_ and 925 μl double‐distilled water in a reaction tube. Fifty microliter (50 μl) ferrozine solution (5 mM) was added and mixed thoroughly. The mixture was then allowed to stand at room temperature for 10 min, and a 200 μl was pipetted into a clear bottom 96‐well plate. A control was also conducted by replacing the sample with 500 μl of double‐distilled water. The absorbance values of control (*A*_*c*_) and sample (*A*_*s*_) at 562 nm were measured using a spectrophotometer. Percentage chelating effect (%) was calculated using the following equation:

$$\text{Metal chelating activity}\left(\%\right)=\frac{A_{c}-A_{s}}{A_{c}}\times 100$$

#### 2.5.4 Determination of hydroxyl radical scavenging assay

The hydroxyl radical scavenging assay (HRSA) was modified based on a method described by Girgih et al. (2011). Sample or GSH (final assay concentration of 1 mg/ml) and 1, 10‐phenanthroline (3 mM) were each separately dissolved in 0.1 M phosphate buffer (pH 7.4) while FeSO_4_ (3 mM) and 0.01% hydrogen peroxide were each separately dissolved in distilled water. An aliquot (50 μl) of sample or GSH (equivalent to a final assay concentration of 1 mg/ml) or buffer (control) was first added to a clear, flat‐bottom 96‐well plate followed by additions of 50 μl of 1, 10‐phenanthroline and 50 μl of FeSO_4_. To initiate the Fenton's reaction in the wells, 50 μl of hydrogen peroxide (H_2_O_2_) solution was added to the mixture, which was then covered. Thereafter, the absorbance of the mixtures was measured at 536 nm every 10 min for a period of 1 hr at 37°C with shaking. The hydroxyl radical scavenging activity was calculated as follows based on changes in absorbance (ΔA):

HRSA(%)=(ΔA/min control−ΔA/min sample)/(ΔA/min control)×100

#### 2.5.5 Statistical analyses

Triplicate readings of the results were taken and analyzed with SPSS version 22 while the means were separated with Duncan's multiple range (DMR) test at *p* < 0.05.

## 3 RESULTS AND DISCUSSION

### 3.1 DPPH radical scavenging capacities

**Figure 1:**
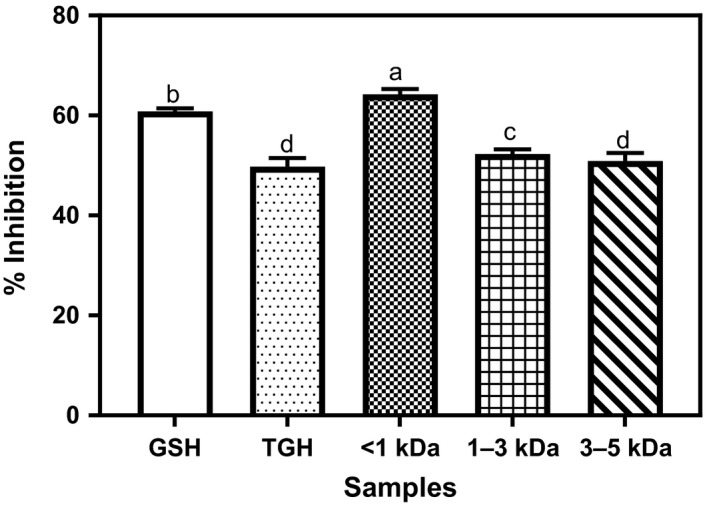
Percentage DPPH radical scavenging capacities of *Moringa oleifera* seed protein hydrolyzate and its membrane fractions. Bars (means ± standard deviation) with different letters are significantly different at *p* < 0.05

DPPH is a stable free radical compound with a proton‐accepting tendency from other donating compounds such as antioxidants (Karadag, Ozcelik, & Saner, 2009; Zhu, Qiu, & Yi, 2010). The free radical scavenging abilities of TGH and its membrane fractions are shown in Figure 1. The <1 kDa TGH was the most active (64.24%) among all the samples. It showed better scavenging ability (*p* < 0.05) than the positive control, GSH (60.83%). Membrane fractionation led to improved activities in all the fractions though there was no statistically significant difference between the 50.92 and 49.75% obtained for 3–5 kDa peptide fraction and the unfractionated hydrolyzate (TGH), respectively. The better free radical scavenging ability of the <1 kDa when compared to other fractions is similar to what was observed in previous studies (Girgih et al., 2011; He, Girgih et al., 2013). The results obtained in this study (64.24%) for <1 kDa is higher compared to the 24.2% and 42% reported for hemp seed and *Dioclea reflexa* seed protein fractions, respectively (Akinyede et al., 2016; Girgih et al., 2011). The 49.75% inhibition shown by the hydrolyzate is also moderately significant when compared to very low (4%) inhibition reported by Girgih et al. (2011). Similarly, in other studies, the hydrolyzate was observed to show no detectable activity against the DPPH radicals (Akinyede et al., 2016; Mundi & Aluko, 2014). This may confirm earlier observation that the radical scavenging ability of food protein hydrolyzate depends on such factors as the hydrolyzing enzyme specificity, amino acid composition and the size of the peptides, and the conditions of DPPH test (Udenigwe, Lu, Han, Hou, & Aluko, 2009). In summary, the improved DPPH radical scavenging ability of the TGH fractions may be due to their higher electron‐donating tendency thereby forming stable, nonreactive radicals through radical‐peptide condensation (Xiong, 2010).

### 3.2 Ferric reducing antioxidant power (FRAP)

**Figure 2:**
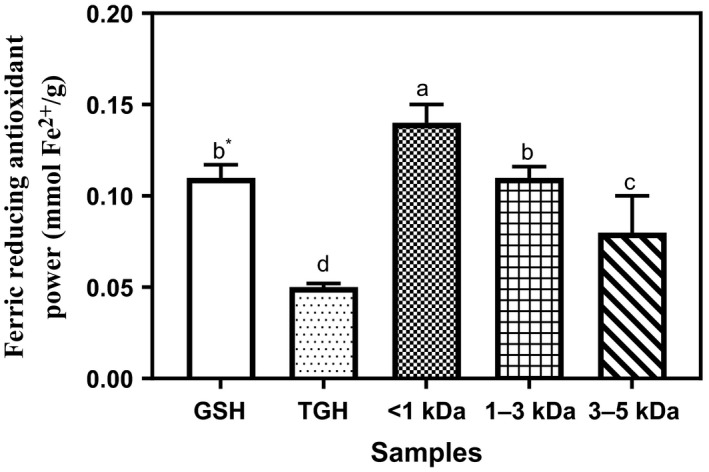
Ferric reducing antioxidant power of *Moringa oleifera* seed protein hydrolyzate and its membrane fractions. Bars (means ± standard deviation) with different letters are significantly different at *p* < 0.05

The FRAP is an assay used to measure the electron‐donating abilities of natural antioxidants. Previous studies have shown that there exists a direct correlation between antioxidant abilities and the reducing power of protein hydrolyzates and peptide fractions (H.‐M. Li et al., 2009; Tang et al., 2012). Figure 2 shows the FRAP results of TGH and peptide fractions. The peptide fractions showed significantly better FRAP abilities (0.08–0.14 mmol Fe^2+^/g) compared to the unfractionated TGH (0.05 mmol Fe^2+^/g). The lower potency of the TGH may be due to dilution effect, that is, higher ratio of inactive components to the active components. However, the positive control, GSH at one‐eighth (0.125 mg/ml) of the samples concentration (1 mg/ml), still showed 79% (0.11 mmol Fe^2+^/g) ferric reducing ability when compared to the best performing <1 kDa (0.14 mmol Fe^2+^/g). Higher ferric reducing ability of GSH compared to protein hydrolyzates or peptide fraction had also been reported in some previous studies (Akinyede et al., 2016; Girgih et al., 2011; He, Girgih et al., 2013). Also, the higher FRAP of the <1 kDa peptide fraction when compared to other fractions is similar to the earlier observed trends in previous studies (Ajibola et al., 2011; Akinyede et al., 2016; Cumby et al., 2008; He, Girgih et al., 2013). The FRAP of proteins or peptide fractions had been reported to be dependent on specificity of hydrolyzing enzyme (He, Girgih et al., 2013). Ma and Xiong (2009) confirmed this when they obtained a negative FRAP activity for pepsin‐digested buckwheat proteins but later obtained activity on subsequent inclusion of pancreatin to the pepsin digest.

### 3.3 Hydroxyl radical scavenging abilities (HRSA)

**Figure 3:**
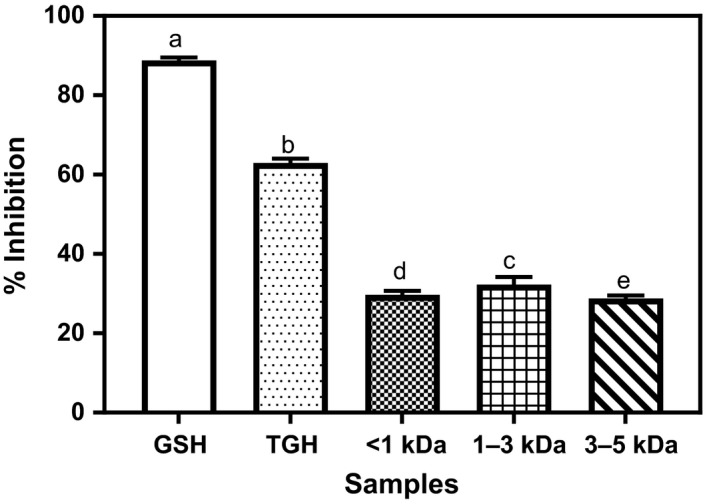
Percentage hydroxyl radical scavenging capacities of *Moringa oleifera* seed protein hydrolyzate and its membrane fractions. Bars (means ± standard deviation) with different letters are significantly different at *p* < 0.05

The HRSA of TGH and its peptide fractions are shown in Figure 3. The positive control, GSH, showed significantly higher (*p* < 0.05) (89%) HRSA compared to TGH and its peptide fractions. These results also showed that TGH possessed significantly higher (63%) HRSA when compared to the 32% of the best performing fraction (1–3 kDa). This is contrary to the reports of Akinyede et al. (2016) and Girgih et al. (2011) where the hydrolyzate showed lower (<10%) and no detectable HRSA potentials, respectively, when compared to the peptide fractions. The observed difference may be due to different protein source, type and specificity of hydrolyzing enzyme used, and processing and assay conditions the samples were subjected to. The higher HRSA of TGH may be due to additive effect in the unfractionated protein hydrolyzate. The 32% obtained in this current study for 1–3 kDa peptide fraction is higher than 24% and 22% reported for hemp seed and *D. reflexa* seed protein fractions, respectively (Akinyede et al., 2016; Girgih et al., 2011), but comparable to 28% obtained for African yam bean protein fraction (Ajibola et al., 2011). In summary, with higher HRSA of TGH, it may offer better HRSA compared to its peptide fractions.

### 3.4 Metal chelation ability (MCA)

**Figure 4:**
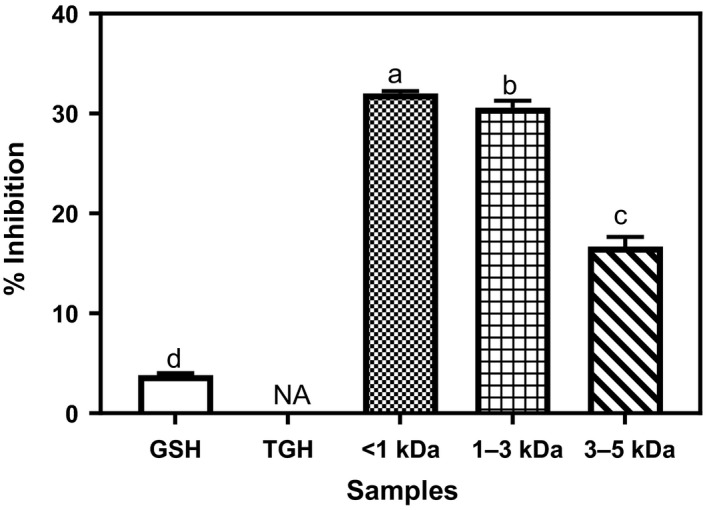
Percentage metal chelation abilities of *Moringa oleifera* seed protein hydrolyzate and its membrane fractions. Bars (means ± standard deviation) with different letters are significantly different at *p* < 0.05. NA: no activity

The MCA abilities of TGH and its membrane fractions are shown in Figure 4. TGH showed no detectable metal chelation ability. This is contrary to the 72% and about 35% obtained for hemp seed and *D*. *reflexa* seed protein hydrolyzates (Akinyede et al., 2016; Girgih et al., 2011). Significantly improved (*p* < 0.05) activities (17%–32%) were however displayed by the fractions after membrane fractionation. However, the values obtained in the current study is lower than the between 70% and 90% obtained for *D. reflexa* (Akinyede et al., 2016) but comparable to 16%–39% obtained for hemp seed protein fractions (Girgih et al., 2011) and higher than the between 15% and 20% obtained for kidney bean protein hydrolyzate (Mundi & Aluko, 2014). The positive control, GSH, showed very low (4%) MCA. The low MCA of GSH when compared to peptide fractions had also been observed in previous studies (Akinyede et al., 2016; Xie, Huang, Xu, & Jin, 2008). According to Mundi and Aluko (2014), iron is essential in promoting oxidative stress. Thus, the formation of nontoxic metal complexes through the binding of free redox‐active iron from its active sites by chelating agents (e.g., bioactive peptides) could be essential in preventing oxidative injury. The peptide fractions, especially, the <1 kDa, may be a good preservative by serving as a metal chelating agent in food system to prevent metal‐ion‐catalyzed reactions which may cause oxidative damages to the food lipids (Akinyede et al., 2016).

### 3.5 Inhibition of angiotensin‐converting enzyme (ACE)

**Figure 5:**
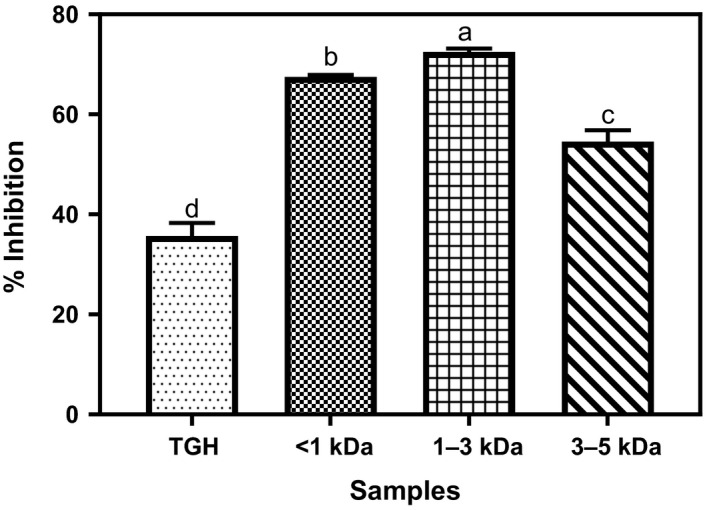
Percentage angiotensin‐converting enzyme inhibition of *Moringa oleifera* seed protein hydrolyzate and its membrane fractions. Bars (means ± standard deviation) with different letters are significantly different at *p* < 0.05

TGH and its membrane fractions showed varied ACE inhibitory potentials as shown in Figure 5. The activity of TGH (36%) is significantly lower (*p* < 0.05) than that of the peptide fractions (55%–72%). The 36% ACE inhibition obtained in this study is lower than the value (>77%) obtained for kidney bean protein hydrolyzate (Mundi & Aluko, 2014) but higher than 23.62% obtained for mung bean protein hydrolyzate (G. H. Li et al., 2005). Lower peptide size may have accounted for the higher ACE inhibitory ability of the peptide fractions when compared to the bigger molecular size of the unfractionated TGH. This may also be responsible for the better performance of the <1 and 1–3 kDa fractions compared to the 3–5 kDa. Lower potency of hydrolyzate against ACE when compared to peptide fractions was also reported for rapeseed protein hydrolyzate (He, Alashi et al., 2013). The results of the current study also agree with previous studies which attributed higher ACE inhibitory potentials to low molecular weight peptide fractions (Campos, Guerrero, & Ancona, 2010; He, Alashi et al., 2013; Zhu et al., 2010). However, further in vivo studies may be necessary to confirm the observed ACE inhibitory ability observed in this study. Reports from previous studies have indicated that in vitro ACE activities may not necessarily translate to antihypertensive activity in vivo because ACE inhibitory peptides must be absorbed in the intestine in their intact state and be resistant to further degradation by plasma peptidases to reach their target sites after oral administration or intravenous infusion in order to exert their antihypertensive properties (Li et al., 2004; Li et al., 2005).

### 3.6 Inhibition of renin

**Figure 6:**
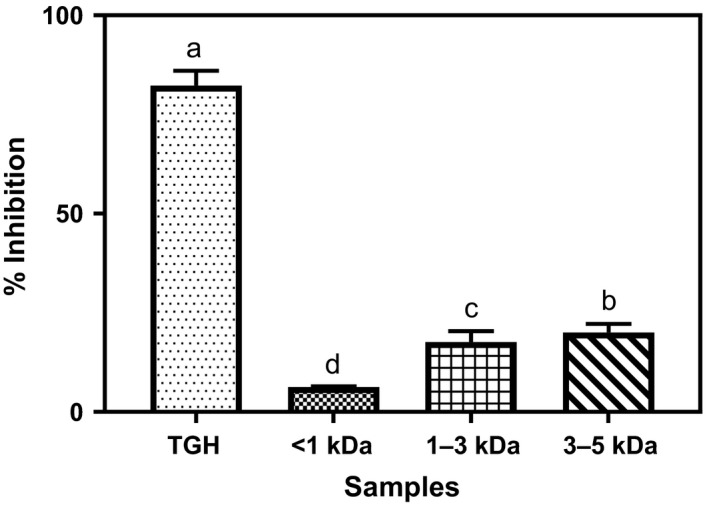
Percentage renin inhibition of *Moringa oleifera* seed protein hydrolyzate and its membrane fractions. Bars (means ± standard deviation) with different letters are significantly different at *p* < 0.05

Renin inhibitory potential of TGH and its fractions is shown in Figure 6. Unlike the ACE inhibition ability of the samples where the membrane fractions showed better inhibition when compared to the unfractionated hydrolyzate, an opposite effects were obtained for the renin inhibition. As shown in Figure 6, TGH is clearly significantly better than its membrane fractions. Again, among the peptide fractions, it was observed that the potency against renin increased as the molecular size of the peptide fraction increased. This report however differs from that of Mundi and Aluko (2014) where membrane fractionation led to improved renin inhibition over the precursor hydrolyzate. Indirect relationship in potency against ACE and renin, that is sample with higher potency against ACE showing lower potency against renin, was also observed in previous studies (Ajibola et al., 2013; Udenigwe et al., 2009; Yuan, Wu, & Aluko, 2007). The value (82%) obtained for TGH in the study is higher than the 45% and 55% reported for flaxseed and African yam bean peptide fraction, respectively (Ajibola et al., 2013; Udenigwe et al., 2012)**.**

## 4 CONCLUSION

Results from this study have shown the potential of free radical scavenging abilities of trypsin‐derived *Moringa oleifera* seed globulin and its membrane fractions. Membrane fractionation produced peptide fractions with improved radical scavenging (DPPH), and ferric reducing and metal (iron) chelating abilities. The peptide fractions also showed high potency against angiotensin‐converting enzyme. While trypsin‐derived hydrolyzate and it peptide fraction could serve as potential antioxidant agents to prevent lipid oxidation in food systems, further studies on purification and in vivo investigations are necessary to establish the observed in vitro ACE‐renin inhibition.

## CONFLICTS OF INTEREST

The authors declare that there are no conflict of interests in the publication of the manuscript.

## ETHICAL REVIEW

This study does not involve any human or animal testing.
